# Machine learning to improve the understanding of rabies epidemiology in low surveillance settings

**DOI:** 10.1038/s41598-024-76089-3

**Published:** 2024-10-28

**Authors:** Ravikiran Keshavamurthy, Cassandra Boutelle, Yoshinori Nakazawa, Haim Joseph, Dady W. Joseph, Pierre Dilius, Andrew D. Gibson, Ryan M. Wallace

**Affiliations:** 1 Poxvirus and Rabies Branch, Division of High Consequence Pathogens and Pathology, National Center for Emerging and Zoonotic Infectious Diseases, Centers for Disease Control and Prevention, Atlanta, GA USA; 2 Ministère de l’Agriculture, des Ressources Naturelles et du Développement Rural, Port au Prince, Haiti; 3 Mission Rabies, Cranborne, Dorset UK

**Keywords:** Rabies epidemiology, Prediction, Machine learning, Extreme gradient boosting, Risk stratification, Zoonotic disease surveillance, Viral infection, Statistics, Machine learning, Predictive medicine, Statistical methods

## Abstract

In low and middle-income countries, a large proportion of animal rabies investigations end without a conclusive diagnosis leading to epidemiologic interpretations informed by clinical, rather than laboratory data. We compared Extreme Gradient Boosting (XGB) with Logistic Regression (LR) for their ability to estimate the probability of rabies in animals investigated as part of an Integrated Bite Case Management program (IBCM). To balance our training data, we used Random Oversampling (ROS) and Synthetic Minority Oversampling Technique. We developed a risk stratification framework based on predicted rabies probabilities. XGB performed better at predicting rabies cases than LR. Oversampling strategies enhanced the model sensitivity making them the preferred technique to predict rare events like rabies in a biting animal. XGB-ROS classified most of the confirmed rabies cases and only a small proportion of non-cases as either high (confirmed cases = 85.2%, non-cases = 0.01%) or moderate (confirmed cases = 8.4%, non-cases = 4.0%) risk. Model-based risk stratification led to a 3.2-fold increase in epidemiologically useful data compared to a routine surveillance strategy using IBCM case definitions. Our study demonstrates the application of machine learning to strengthen zoonotic disease surveillance under resource-limited settings.

**Supplementary Information:**

The online version contains supplementary material available at 10.1038/s41598-024-76089-3.

## Introduction

Rabies is a fatal viral zoonosis estimated to be responsible for over 70,000 human deaths every year^1,2^. Rabies virus is transmitted mainly through the bite of an infected animal with 99% of all human deaths attributed to bites from domesticated dogs^3^. The disease mostly affects marginalized segments of society, most of whom belong to low and middle-income countries (LMICs) in Asia and Africa^4,5^. Rabies is a preventable disease when post-exposure prophylaxis (PEP) is promptly administered. However, inadequate availability of PEP at healthcare facilities and lack of public awareness regarding rabies prevention have resulted in persistent human rabies deaths. Despite a high burden of rabies in LMICs, patients rarely seek healthcare or undergo diagnostic testing, resulting in fewer than 5% of likely cases reported to health authorities^6–8^. The global rabies community has set an ambitious goal to reach zero dog-mediated human rabies deaths by 2030. To reach this goal, drastic measures must be taken to improve surveillance systems so that they can accurately monitor disease, inform interventions, advocate for effective control measures, and verify when elimination has been achieved.

Ideally, surveillance would be powered by the detection of animal rabies cases through gold-standard laboratory diagnostic assays, as defined by the World Organization for Animal Health (WOAH)^9^. These tests have undergone extensive validation to demonstrate high viral sensitivity and specificity. Highly sensitive tests are necessary to avoid false-negative results, which could result in exposed persons and animals not seeking life-saving medical care. High specificity is particularly important as the cost for human rabies vaccine is often equivalent to one month’s salary in many LMICs^10^. However, many rabies-endemic communities lack ready-access to laboratory testing services, and while an increase in laboratory capacity is necessary for a strong surveillance system, this must also be accompanied by an increase in the quality and quantity of field-based rabies case investigations.

Investigation-based surveillance has the potential to progress rabies programs in the absence of decentralized laboratory capacity, although current clinical-based case definitions may not provide desired specificity. After a suspected rabies exposure, the World Health Organization (WHO) recommends a standardized investigation of the offending animal as part of a comprehensive risk assessment to inform human PEP decisions^11^. To support healthcare provider risk-assessment decisions, WHO has defined four case definitions: non-cases, suspected cases, probable cases, and confirmed cases^11^. To minimize the risk of human deaths, the WHO probable case definition (also referred to as a “clinically confirmed” case) is highly sensitive and includes any biting animal that is unavailable for a health assessment at 10 days after the exposure; rabies virus shedding can be ruled out in dogs, cats, and ferrets that remain healthy 10 days after the exposure. While this approach works well for ensuring those with true rabies exposures get life-saving medical care, the low specificity of the WHO definition of a clinical rabies case may not be suitable for epidemiological monitoring, as it could overestimate the true rabies burden at the population level, confound epidemiological interpretations, and stifle effective interventions. This strategy may also result in incurring unnecessary expenses on surveillance programs to investigate animals that are unlikely to pose a rabies risk, potentially limiting the availability of resources to the communities in most need.

WHO recommends that countries endemic for dog-mediated rabies invest in advanced surveillance systems, which include the basic principles of (a) a system to detect and document dog bite exposures, (b) veterinary assessment of biting dogs and other animals suspected of rabies, (c) human rabies vaccination decisions informed by a situational risk assessment^11^. A primary challenge of implementing this recommendation stems from the high rate of dog bites (up to 3% of the human population each year), low rate of rabies in biting dogs (estimated at 1% or less among all biting dogs), and limited veterinary capacity for routine assessment of biting dogs^12–14^. As a result, veterinary professionals must prioritize which events to investigate, often leading to incomplete epidemiologic and laboratory-based data. An expansion of investigation-based surveillance with a highly sensitive and specific framework is necessary to progress towards zero dog-mediated human rabies deaths and would present the lowest barrier to drastically improving surveillance in LMICs.

Logistic regression (LR) is the commonly used technique to predict rabies in biting dogs^15,16^. However, traditional modeling techniques like LR have strict assumptions for data distributions which limit their ability to handle large and complex epidemiological data^17^. In the past decade, modern machine learning (ML) techniques have gained popularity because of their ability to detect complex non-linear relationships between dependent and independent variables^18,19^. In this study, we highlight modeling approaches to predict animal rabies, in real-time, based on historical and clinical data mostly collected during rabies investigations in Haiti. We use advanced machine learning and data processing techniques to increase overall predictive performance. We create a risk stratification system to prioritize case investigations based on predicted rabies probabilities in settings with limited surveillance and diagnostic resources.

## Materials and methods

### Rabies surveillance data

Integrated Bite Case Management (IBCM) is a form of advanced rabies surveillance recommended by WHO to reduce human and animal rabies mortality^11^. Haiti’s national rabies surveillance program was established in 2013 using paper-based IBCM and was upgraded to an electronic smartphone application in 2018^20^. The electronic data collected during animal rabies investigations using the Rabies Exposure Assessment and Contract Tracing (REACT) app between June 2018 and November 2023 was used in this study. Detailed information about the data collected using the REACT app in Haiti is presented elsewhere^20^. The dataset consisted of information related to the circumstances of the rabies exposure, animal health assessment conducted by government para-veterinarians, case investigation outcomes, and laboratory testing (when applicable). From 2018 to 2021, diagnostic testing was performed by the Haiti National Rabies Laboratory, which utilizes the Direct Fluorescent Antibody Test. After 2021, due to social instability and loss of laboratory capacity, CDC implemented a lateral flow test for diagnostic testing; Haiti partners with the US Centers for Disease Control’s WOAH Rabies Reference Laboratory to conduct routine inter-laboratory testing comparisons and selective confirmatory testing.

After rabies investigations, animals were classified into one of four categories; confirmed, probable, suspect, and non-case using Haiti’s IBCM case definition, in alignment with the WHO clinical case definition with minor modification (Table 1). The information related to potentially rabid animals was used as input features for prediction models (Table 2).

**Table 1:** The IBCM case definition adapted to assign the status of investigated animal rabies cases.

Case status	WHO	Haiti IBCM
**Suspect**	A case that is compatible with a clinical case definition of animal rabies: • Hypersalivation • Paralysis • Lethargy • Unprovoked abnormal aggression (biting two or more people or animals and/or inanimate objects) • Abnormal vocalization • Diurnal activity of nocturnal species	Animals that had one or more signs of rabies **OR** Animals reported for suspicion of rabies, but no additional information was available **AND** Did not pass observation (escaped animals, those not found for assessment) **AND** Were not known to have died within 10 days
**Probable**	A suspected case **AND** A reliable history of contact with a suspected, probably or confirmed rabid animal **OR** Is killed, died or disappears within 4–5 days of observation of illness	Developed one or more clinical signs consistent with rabies **AND** Animals that were not tested for rabies or test results were inconclusive **AND** Died/killed during the 10-day observation period
**Confirmed**	Diagnostic confirmation of rabies virus	Diagnostic confirmation of rabies virus
**Not a case**	A suspected or probable case that is ruled out by laboratory tests **OR** Epidemiological investigation (i.e. appropriate quarantine period in eligible animals).	Animals that tested negative **OR** Animals that are healthy after the 10-day observation period

**Table 2:** Description of model input features collected during routine IBCM investigations.

Feature	Categories
Age	Infant/Junior/Adult/Unknown
Vaccination status	Yes/No/Unknown
Species	Dog/Cat/Others
Dead during investigation	Yes/No or not reported
Bit two or more people/animals	Yes/No or not reported
Hypersalivation	Yes/No or not reported
Paralysis	Yes/No or not reported
Lethargy	Yes/No or not reported
Vocal changes	Yes/No or not reported
Aggression	Yes/No or not reported

### Data balancing

The relatively low rate of rabies among IBCM case investigations (typically < 5%) can lead to prediction inaccuracies, as models would have fewer chances to learn from these minority events. To address this, we assessed two data oversampling techniques: Random oversampling (ROS), and Synthetic Minority Oversampling Technique (SMOTE).

ROS involves generating new samples by randomly selecting underrepresented (minority) class observations and adding them to the training dataset. This oversampling process compensates for the class imbalance by biasing the discrimination process. SMOTE generates synthetic samples from the minority class by interpolating existing observations^21^. The sample point is generated using the K nearest neighbors (KNN) algorithm by calculating the distance value between feature vectors and their closest neighbors in the minority class^22^. ROS and SMOTE balancing methods were implemented using the “imblearn” package in Python 3.11.7. The ROS and SMOTE were applied to training data until the underrepresented rabies-positive class was equal to that of the rabies-negative class. The rabies prediction models were built on both imbalanced and balanced training data to compare their effects on model performance.

### Rabies prediction models

We used two classification algorithms, Logistic Regression (LR) and Extreme Gradient Boosting (XGB) for predicting investigation outcomes using case history and symptomatic data. The LR and XGB models were built using the “sklearn” and “XGBoost” packages, respectively in Python 3.11.7.

The LR is a fundamental and efficient classification model that uses logistic functions and a maximum likelihood estimator for its implementation. It could be used for both binary classification and probability estimation. In our study, LR provided a benchmark to compare the performance of XGB and data-rebalancing techniques. The variable inflation factors were used to identify multicollinearity and only the features with values less than 5 were retained in the final model.

The XGB model is a powerful ensemble machine-learning algorithm used for both classification and regression problems^23^. This technique uses a scalable implementation of the gradient-boosting framework to produce hundreds of low-accuracy decision trees and combines them into a single highly accurate ensemble model. The grid search technique with a 5-fold cross-validation split was performed to identify the optimal combination of hyperparameters to be used for the XGB classification model. The feature importance scores based on feature weights were estimated to understand the effect of each input variable on the rabies outcome.

### Probability calibration

Most predicted probabilities, especially from ML classifiers are not calibrated to reflect the observed proportions in real-world scenarios resulting in a discrepancy between a prediction and the actual frequency^24^. Additionally, data rebalancing techniques including ROS and SMOTE produce consistently biased probability estimates. We applied isotonic regression^25^ with a 5-fold cross-validation using the “sklearn” package to correct potentially biased probability estimates of all the model-sampling technique combinations.

### Model evaluation

We evaluated the performance of rabies prediction based on the following metrics.

- Threshold metrics: Sensitivity (SN), Specificity (SP), and Accuracy (AC).
- Rank metrics: Precision-Recall Area Under the Curve (PR-AUC) and Receiving Operating Characteristic Area Under the Curve (ROC-AUC).
- Probability metrics: Brier score (BS).

The probability of a case investigation concluding in a confirmed rabies case was predicted as model output for each rebalancing technique and prediction model combination. The predicted probabilities that animals under investigation had rabies were in the range [0, 1]. The evaluation was conducted for a range of rabies probability threshold values (0.01 to 1). i.e., the animal was classified as rabies-positive if its predicted probability was above a specified threshold value. A confusion matrix containing true positive (TP), true negative (TN), false positive (FP), and false negative (FN) predictions was obtained for each cutoff. Threshold metrics i.e., SN, SP, and AC were calculated (Supplementary Table S1). Special emphasis was given to maximizing model SN while maintaining reasonably high SP and AC to minimize the risk of false negative classification of rabies cases.

Rank metrics such as PR-AUC and ROC-AUC evaluate models based on their ability to differentiate and correctly classify investigation outcomes for non-cases and confirmed cases. Evaluating models built on imbalanced datasets using the ROC-AUC results in over-optimistic estimates. Hence, PR-AUC was preferred to select the best-performing model in our study as it prioritizes minority class more compared to ROC-AUC^26^.

Probability metrics help better understand how well-calibrated models are. We used the Brier score (BS) for probability evaluation (Supplementary Table S1). The BS reflects the models’ ability to produce rabies probabilities that match observed proportions in real-world scenarios. Smaller BS indicates better performance.

### Risk stratification

**Fig. 1:**
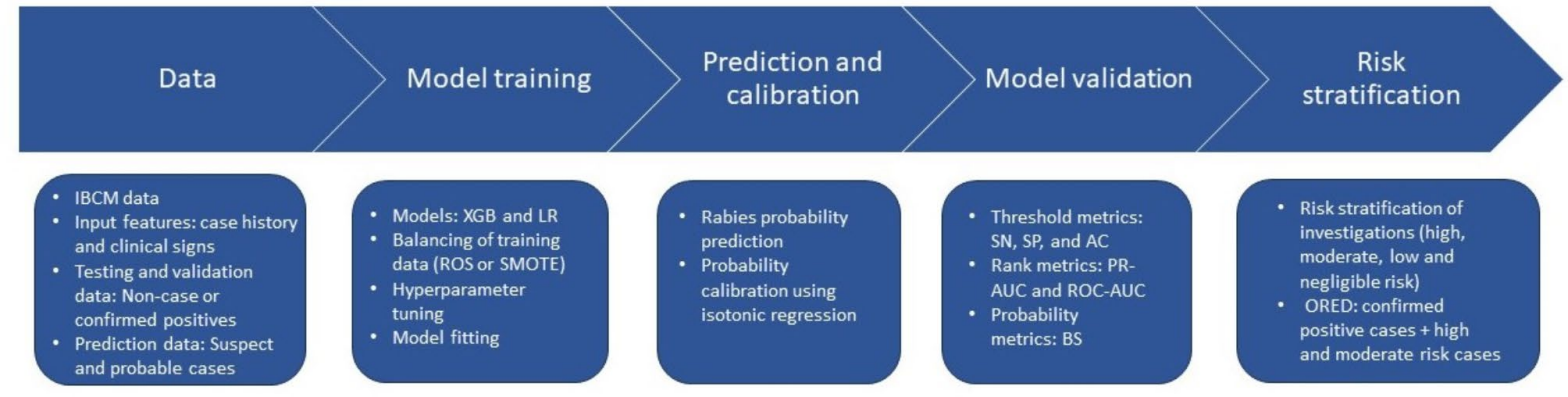
Flowchart illustrating various steps in building and analyzing rabies prediction models.

We grouped predicted rabies probabilities by their IBCM investigation status as defined in Box 1. The median and interquartile ranges of the predicted rabies probabilities for each of these IBCM investigation statuses were calculated. Finally, we used a flexible threshold scheme to stratify the rabies probabilities into four risk categories: high risk, moderate risk, low risk, and negligible risk, which were compared to WHO and IBCM classifications: confirmed, probable, suspect, and non-case. Finally, we created an Optimized Rabies Epidemiologic Dataset (ORED) containing all confirmed positive cases along with model-derived high and moderate-risk cases, excluding cases that were confirmed negative or passed 10-day observation period (e.g., non-cases). This database was created to maximize the epidemiological usability of the animal rabies surveillance data collected during routine IBCM investigations, to account for cases likely to be rabid, but lacking a confirmatory investigation outcome. The graphical illustration of the various steps involved in building and analyzing rabies prediction models is presented in Fig. 1.

## Results

Between June 2018 and November 2023, a total of 13,073 investigations were recorded in the REACT app of which only 6,326 (48.4%) had a final case status of “non-case” or “confirmed”. Overall, 95 animals were confirmed rabies positives, and 6,231 were ruled out for rabies either through testing (1.5%) or passing a 10-day post-bite observation period (98.5%). The data belonging to these investigations were used to train and evaluate the prediction model. For the remaining investigations that lacked final case status, the rabies probabilities were predicted using the best-performing model.

### Model evaluation

Since a large proportion (98.5%) of case investigations with a definitive outcome were non-cases, we used ROS and SMOTE to rebalance the training data used for our modeling. The evaluation metrics for each prediction model and rebalancing technique combinations at 0.5 predicted probability cutoff are presented in Table 3. Overall, XGB had better predictive performance compared to LR. The ROS and SMOTE were better at maximizing SN which came at the cost of a slight reduction in AC and PR-AUC compared to their respective base models trained with imbalanced data. The XGB-SMOTE and XGB-ROS had the highest SN of 0.95 while maintaining acceptable SP, AC, and PR-AUC.

**Table 3:** The evaluation metrics for rabies prediction models. The predicted probability cutoff of 0.5 was used to obtain rabies-positive and negative predictions for validation data.

Model	SN	SP	AC	PR-AUC	ROC-AUC	BS
LR	0.65	0.99	0.99	0.65	0.82	0.009
LR-ROS	0.85	0.95	0.94	0.62	0.96	0.026
LR-SMOTE	0.90	0.94	0.94	0.58	0.95	0.035
XGB	0.75	0.99	0.99	0.72	0.87	0.008
XGB-ROS	0.95	0.97	0.97	0.66	0.96	0.030
XGB-SMOTE	0.95	0.96	0.96	0.65	0.98	0.032

The change in performance of rabies prediction models and data balancing techniques across a range of predicted probability thresholds is presented in Supplementary Dataset S1. As expected, the SN increased and SP decreased with the increasing probability cutoff. The LR trained with imbalanced data had the least desirable evaluation metrics. The XGB-ROS and XGB-SMOTE achieved high SN while maintaining acceptable SP even with a higher probability cutoff range of 0.5 to 0.7 (Supplementary Dataset S1).

The feature importance plots for XGB, XGB-SMOTE and XGB-ROS models are presented in Supplementary Figure S1. Animals dying within the 10-day observation period, lack of vaccination or unknown vaccination status, and aggression were among the most important factors for animal rabies predictions.

We used isotonic regression to calibrate the predicted rabies probabilities of our models. The BS for both uncalibrated and calibrated models are listed in the Supplementary Table S2. There was no notable improvement in probability metrics after the probability calibration of classifiers. The base models were better calibrated compared to their oversampled counterparts. The calibrated XGB had the best BS (0.008).

Overall, The XGB-ROS and XGB-SMOTE were the models of choice for maximizing sensitivity whereas unbalanced XGB had a better overall performance as indicated by PR-AUC and AC score and produced more reliable probabilities (Supplementary Table S3).

### Risk stratification

**Fig. 2:**
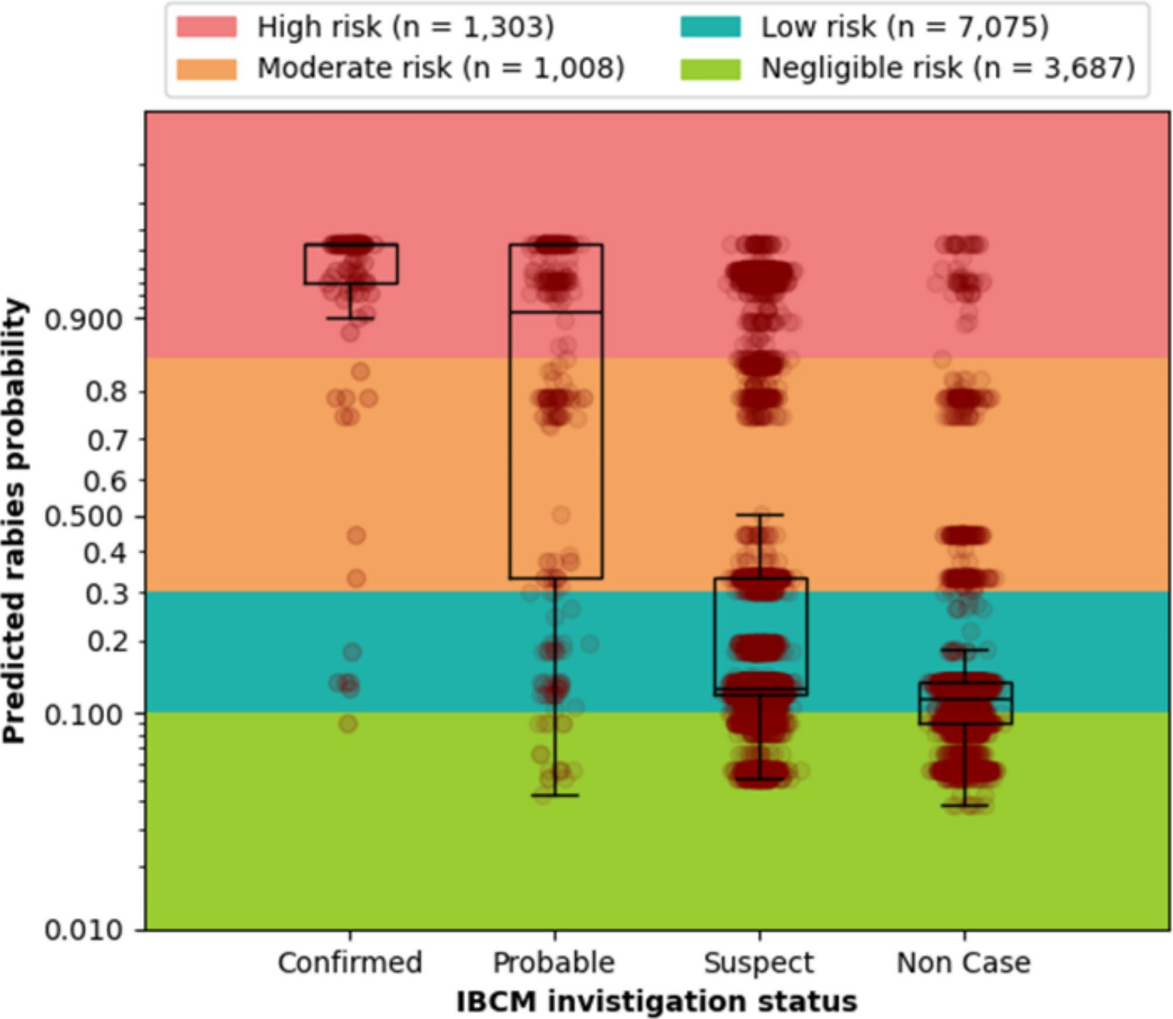
The rabies probabilities of all rabies cases (*n* = 13,073) predicted by XGB-ROS grouped by IBCM investigation status. The rabies probability values of > 0.85, 0.85 − 0.3, 0.29 − 0.1, and < 0.1 were used to classify individual cases into high, moderate, low, and negligible risk categories. The y-axis is shown in the logit scale.

We chose the calibrated XGB-ROS to predict rabies probabilities on all 13,073 investigations recorded through IBCM. The box and whisker plots of predicted rabies probabilities of XGB-ROS grouped by IBCM investigation status are presented in Fig. 2. Cutoff values (i.e., > 0.85, 0.85 − 0.3, 0.29 − 0.1, < 0.1) were used as thresholds to stratify the rabies probabilities of individual cases into high (*n* = 1,303), moderate (*n* = 1,008), low (*n* = 7,075), and negligible (*n* = 3,687) risk categories. Cutoff values were selected based on SN and SP values that minimize the misclassification of confirmed rabies cases into lower-risk categories along with subject matter opinion. The classification of rabies risks by IBCM and WHO case definitions is presented in Table 4. The majority of the confirmed cases and only a small proportion of non-cases were grouped as high (confirmed cases = 85.2%, non-cases = 0.01%) and moderate-risk (confirmed cases = 8.4%, non-cases = 4.0%).

**Table 4:** Classification of rabies risks by IBCM and WHO case definitions. The XGB-ROS model was used for predicting rabies risks.

Status	High Risk	Moderate Risk	Low Risk	Negligible Risk
Confirmed (*n* = 95)	81 (85.2%)	8 (8.4%)	5 (5.3%)	1 (1.1%)
Probable (WHO) (*n* = 1979)	163 (8.2%)	234 (11.8%)	1269 (64.1%)	313 (15.8%)
Probable (IBCM) (*n* = 527)	277 (52.6%)	164 (31.1%)	56 (10.6%)	30 (5.7%)
Suspect (WHO) (*n* = 4768)	1002 (21.0%)	515 (10.8%)	2699 (56.6%)	552 (11.6%)
Suspect (IBCM) (*n* = 6,223)	888 (14.3%)	585 (9.4%)	3912 (62.9%)	835 (13.4%)
Non-Case (*n* = 6,231)	57 (0.01%)	251 (4.0%)	3,102 (49.8%)	2,821 (45.3%)

**Fig. 3:**
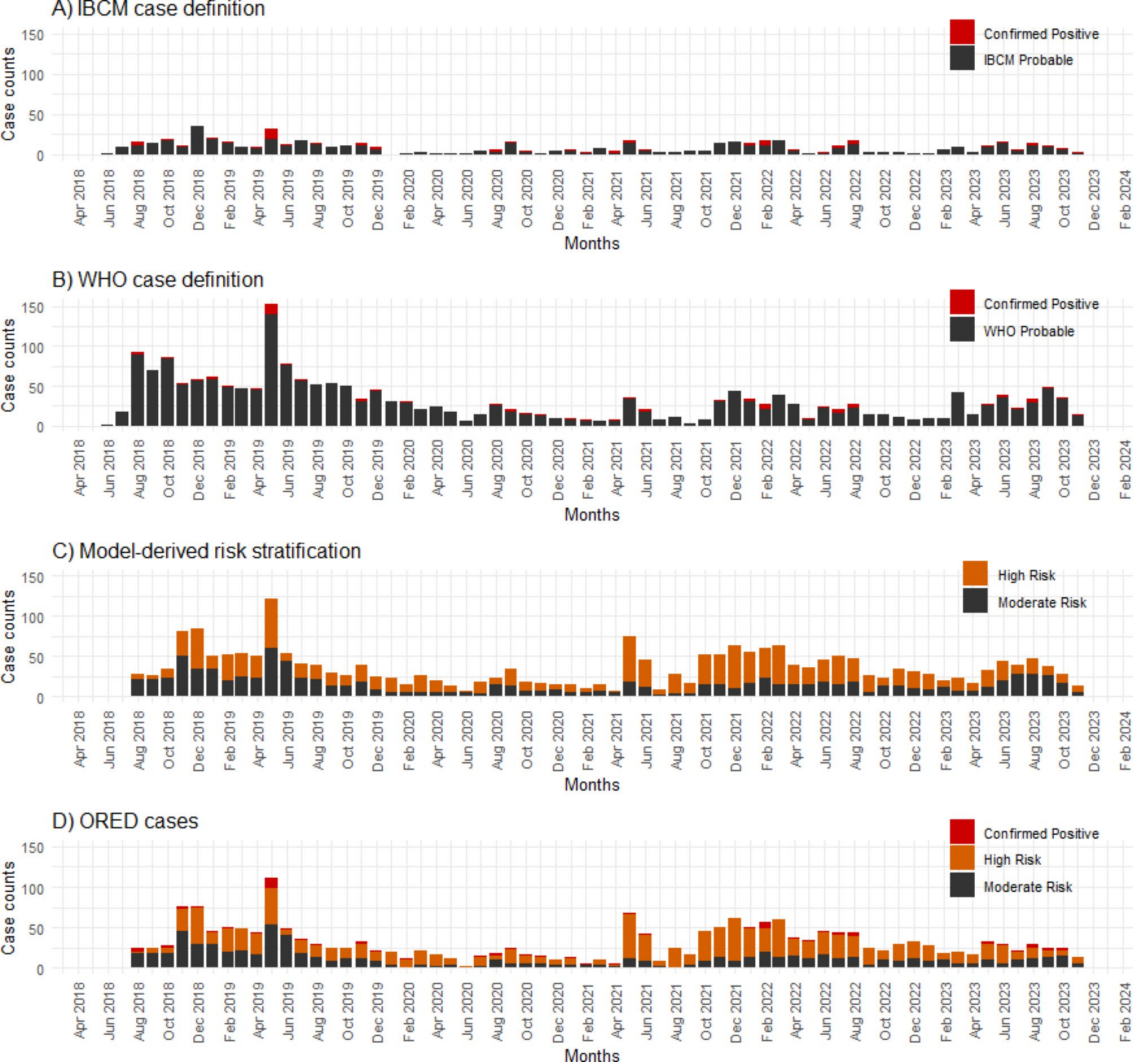
The time series plots of Haiti (June 2018-Nov 2023) depicting monthly reports of investigations. (**A**) IBCM case definition (*n* = 622), (**B**) WHO case definition (*n* = 2,074), (**C**) investigations grouped by model-derived risk stratification (*n* = 2,311), (**D**) Optimized Rabies Epidemiologic Dataset (ORED) cases (*n* = 2,009). The ORED included confirmed rabies positives along with model-derived high and moderate-risk cases and excluded cases that tested rabies negative or passed 10-day observation.

**Fig. 4:**
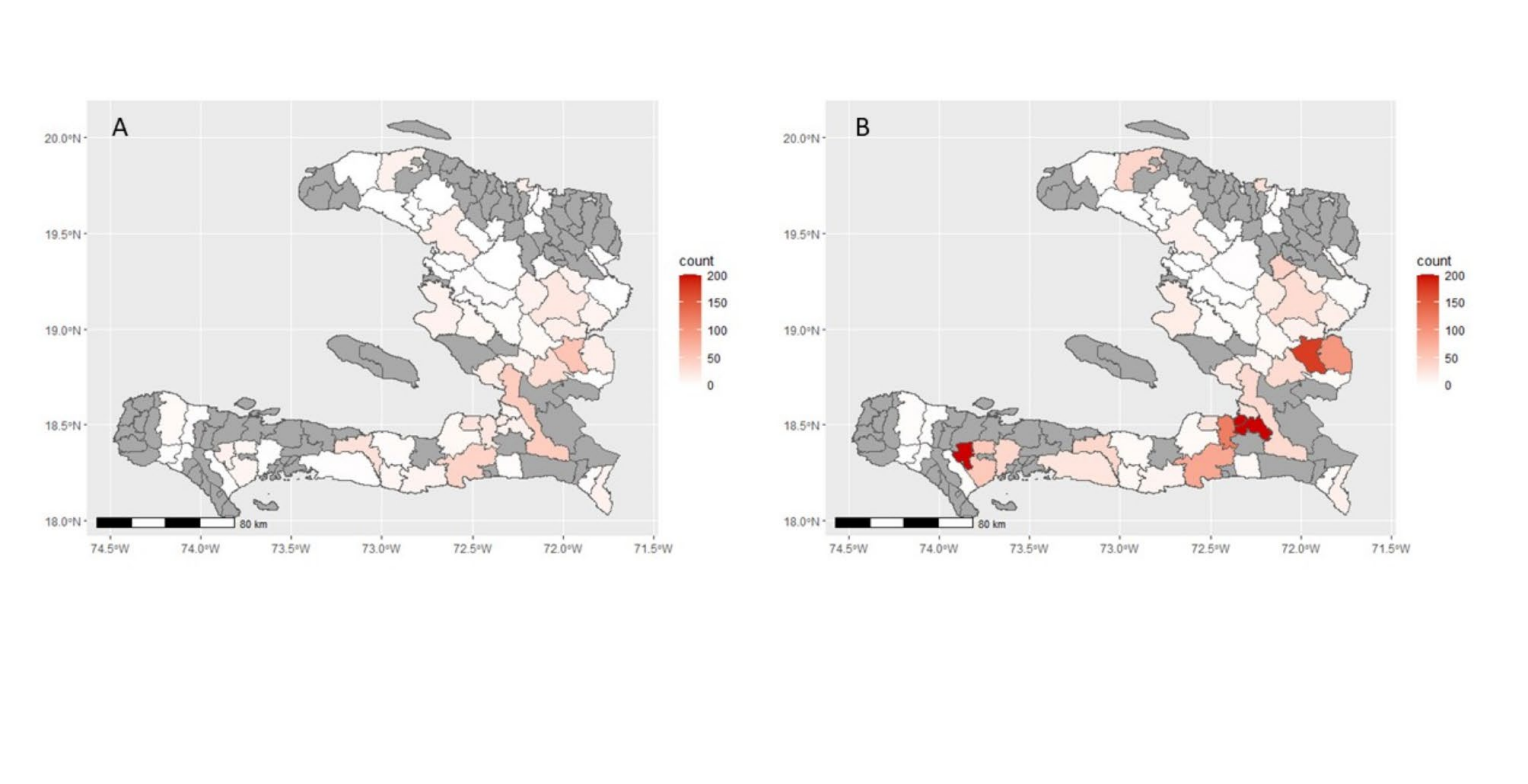
Commune level heat maps of confirmed and likely rabies cases in Haiti (June 2018 - Nov 2023). (**A**) Confirmed or IBCM-probable cases, (**B**) ORED cases that included confirmed rabies positives along with model-derived high and moderate-risk cases and excluded cases that tested rabies negative or passed 10-day observation. The communes with total investigations of less than 10 (gray) were excluded from the heat maps. The maps were generated using R version 4.3.1 (www.r-project.org).

The monthly breakdown of rabies cases based on the WHO, IBCM, and ORED case definitions is presented in Fig. 3. Between June 2018 and Nov 2023, there were 2,009 ORED cases, in contrast to 622 confirmed or IBCM-probable cases, representing a 3.2-fold increase in epidemiological useful data. The ORED and WHO-classified (*n* = 2,074) confirmed and probable cases were similar, however, the WHO classification of “probable” showed low specificity, with only 20% of these cases falling into the high or moderate risk categories. In contrast, the IBCM classification of “probable” aligned well with the risk model, with 83.7% of these cases defined as high or moderate risk (Table 4). Risk mapping of IBCM-classified confirmed and probable cases compared to the ORED cases showed that the latter definition increased the number of communities with rabies detections by 12% (Fig. 4).

## Discussion

This study provides a machine learning-based modeling framework to predict animal rabies outcomes using commonly collected case history and clinical signs from animals that have potentially exposed people to rabies virus. We used the largest available IBCM-derived rabies surveillance dataset from LMICs, which offered standardized, electronically collected data. The data was comprehensive, with a high level of completeness, broad temporal span, and consistent reporting efficiency, resulting in a model with highly accurate and reliable predictions. We also provide a risk stratification framework to prioritize case investigations based on rabies probabilities in settings where resource limitations may prohibit the investigation of all suspected rabies exposures. The prediction approach could increase the usability of case investigation data, particularly in the absence of accessible laboratory services.

In LMICs, a large proportion of animal rabies investigations result in inconclusive outcomes^7^. Often cited barriers for rabies testing and conclusive investigation outcomes include a preponderance for dogs to roam freely, non-compliance from owners for euthanasia, disposal of biting animals, lack of veterinary professionals for assessment and sample collection, lack of sample transportation networks, and lack of available diagnostic facilities^6,27,28^. Hence, in programs that only assess diagnostic results for epidemiologic monitoring, a majority of information collected during rabies surveillance remains largely unusable. Furthermore, there are often great financial costs to operating advanced rabies surveillance systems, making the data collected highly valuable (even those lacking confirmatory outcomes). Introducing a highly sensitive and specific machine-learning model to accurately characterize the risk of probable and suspected rabid animals can maximize the value of these advanced surveillance systems.

Various strategies including monitoring dog bite events, clinically-confirmed rabies cases (based on WHO or project-specific IBCM case definition), and laboratory-confirmed cases have been used as a proxy for tracking rabies trends in regions where surveillance and testing capabilities are limited^29–32^. Reporting and tracking dog bites are an important part of the rabies surveillance system, serving as alerts to initiate field investigations. However, dog bites are poor indicators of rabies trends, as dog bites are a common occurrence and rarely due to a rabid dog^15^. Laboratory testing in many countries presents an incomplete epidemiologic depiction given the many challenges associated with collecting, submitting, and testing samples. Relying solely on laboratory confirmations ignores usable surveillance data and may miss important epidemiologic shifts. When testing capacities are low, risk stratification techniques using highly specific and sensitive rabies prediction models, as demonstrated here, result in a more meaningful epidemiologic understanding of rabies. Furthermore, WHO or IBCM definition based qualitative risk assessments, such as flow chart algorithms, can be complicated when trying to determine if a patient should receive PEP. As modeled risk becomes more commonplace in rabies control programs, quantitative values determining the need for PEP may help streamline rabies exposure treatment decisions.

In our study, a large proportion of animals were classified as WHO-probable, yet only 20% of these aligned with the high and moderate model-derived risk categories, suggesting that this definition likely has low specificity for classifying true rabies cases. The WHO probable case definition is intentionally conservative, making it better suited for PEP recommendations in many settings. Conversely, the IBCM probable case definition is better aligned with model-derived risk categories, suggesting that this is a better option for epidemiologic analysis. The ORED utilizes definitive outcomes (e.g., laboratory confirmation and passing observation periods) followed by model-derived cut-offs when conclusive outcomes are lacking to maximize the usability of surveillance data. Furthermore, ORED increased the usable case data by 3.2-fold and identified 12% additional at-risk communities compared to the IBCM classification scheme. This enhanced understanding of rabies epidemiology could provide a clearer baseline to drive political and philanthropic support for rabies control activities and monitor the impact of nascent dog vaccination campaigns. Machine learning methods could complement epidemiologic monitoring, but as more countries implement WHO recommendations for surveillance, further validations focused on country and program-specific differences should be explored.

Our study used a risk stratification framework with a flexible probability threshold to classify rabies cases into high, moderate, low, and negligible risk events, while also accounting for cases with known outcomes (definitive laboratory or observation outcomes). Such a framework could improve rabies control programs in numerous ways. For instance, with an estimated 3% of people bitten by a dog every year in many countries, there are far too many bite events for even wealthy rabies programs to investigate all offending animals. Based on minimal data typically provided at the time of a bite report, this model could be used to ensure that higher-risk cases are appropriately investigated. Additionally, a probability-based, quantitative risk value ascribed to cases may help convince owners to relinquish high-risk dogs for euthanasia and convince healthcare-averse bite victims to seek much-needed (and potentially costly) treatment. Rather than categorical definitions of “suspect” or “probable”, which have shown to be lacking in statistical accuracy, being able to provide an empirically-derived risk percentage that the animal may have rabies could be another tool for investigators to improve programmatic goals and bite-victim health outcomes.

Despite the relatively low threshold that was used to maximize model sensitivity, a small number of outlier rabies cases were misclassified. Such classification error could be concerning especially when a rabid case is categorized as lower risk. In our analysis, only one confirmed rabid dog was classified as “negligible risk”. This dog had no noted rabies symptoms and had not bitten any people or animals. Similarly, of the five confirmed rabid dogs classified as “low risk”, four had no noted rabies symptoms and only one dog had any documented clinical signs (i.e., aggression). All of these animals were found dead and submitted for testing; WHO recommends PEP in all instances where an animal dies within 10 days of an exposure event. Under any WHO-aligned rabies risk assessment protocol, rabies-exposed victims in these situations would have received PEP. This closer examination of model “failures” is re-assuring in several ways. First, these animals were unlikely to have exposed other people or animals, and based on the model importance parameters, if they had symptoms and had bitten people or animals they would have been classified into a higher-risk category. Second, these findings underscore the importance of rabies programs having clear algorithms for PEP; a quantitative risk score is a supporting factor to consider but should not supersede WHO PEP recommendations.

Our findings show that XGB-based techniques were better at both binary classification and probability estimation compared to LR counterparts. While there was no single best model suitable for all situations, the XBG with oversampling was better at maximizing the model SN while preserving high SP. Oversampling helps counter the unequal outcomes often found in advanced rabies surveillance systems by forcing the prediction models to simulate more rabies outcomes^33^. Hence, models trained with oversampled data typically have improved sensitivity compared to their imbalanced counterparts. However, balancing techniques have an intrinsic tendency to bias the posterior probabilities of classifiers, hindering the goal of having well-calibrated probabilistic classifications^34^. In situations like rabies risk stratification where reliable probabilities are desired, correcting these biases using calibration techniques such as isotonic regression, as performed in this study may be necessary.

Despite rabies being preventable, it remains a neglected disease in most parts of the world in large part due to the lack of adequate surveillance systems to elucidate the true burden in people and animals. There is a wide disparity in the availability of surveillance, testing, and vaccination resources, especially in LMICs. WHO recommends that countries implement advanced surveillance systems to support their efforts to achieve rabies elimination, yet there is little guidance for how programs should utilize the data from these systems. Our study demonstrates the utility of advanced machine learning techniques for accurate and reliable predictions which can help support risk assessment and epidemiologic analysis. However, this approach is likely to be only applicable in settings where advanced surveillance systems have been implemented and the relevance of the specific model presented here to other countries needs further evaluation. Ending the cycle of neglected diseases begins with improved awareness of the burden of the disease. As countries embrace the WHO recommendations to implement advanced surveillance systems, the approach described here will be an integral component of ensuring that these programs maximize the value of their surveillance efforts.

## Electronic supplementary material

Below is the link to the electronic supplementary material.

Supplementary Material 1

Supplementary Material 2

## Data Availability

The datasets used during the current study are available from the corresponding author on reasonable request.
