# Supplementary material for: Machine learning to improve the understanding of rabies epidemiology in low surveillance settings
Source: Sci Rep. 2024 Oct 28;14:25851. doi: 10.1038/s41598-024-76089-3 (PMC11519585; doi:10.1038/s41598-024-76089-3)
Supplement: Supplementary file 1 — Supplementary Material 1 [file 41598_2024_76089_MOESM1_ESM.docx]

**Supporting Information**

Machine learning to improve the understanding of rabies epidemiology in low surveillance settings

Ravikiran Keshavamurthy^1^*, Cassandra Boutelle^1^, Yoshinori Nakazawa^1^, Haim Joseph^2^, Dady W. Joseph^2^, Pierre Dilius^2^, Andrew D. Gibson^3^, Ryan M. Wallace^1^.

* Ravikiran Keshavamurthy

Email: mfl3@cdc.gov


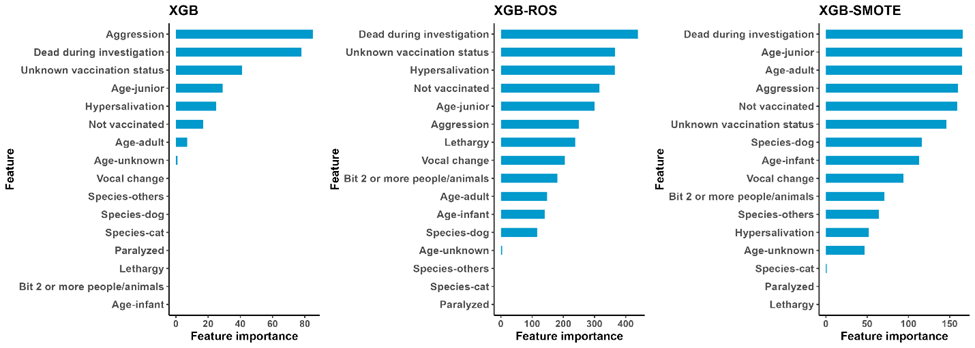


Figure S1. The feature importance plots for XGB, XGB-SMOTE, and XGB-ROS models for animal rabies classification. The information related to case history and clinical signs collected during routine IBCM investigations were used as features.

**Table S1**. Rank and evaluation metrics used for evaluating rabies prediction models.

| **Metrics** | | **Equation** |
| --- | --- | --- |
| Threshold  metrics | Specificity (SP) | $\frac{TN}{FP+TN}$ |
|  | Sensitivity (SN) | $\frac{TP}{TP+FN}$ |
|  | Accuracy (AC) | $\frac{TP+TN}{TP+FP+TN+FN}$ |
| Probability  metrics | Brier score (BS) | $1/N\sum_{t=1}^{N} {(f_{t}-o_{t})}^{2}$  where, f_t_: the classifier output, o_t_: the actual outcome |

Table S2. Brier score for uncalibrated and calibrated rabies prediction models. Smaller metric indicates better calibration.

| **Model** | **Uncalibrated** | **Calibrated** |
| --- | --- | --- |
| **LR** | 0.009 | 0.009 |
| **LR-ROS** | 0.025 | 0.026 |
| **LR-SMOTE** | 0.034 | 0.035 |
| **XGB** | 0.009 | 0.008 |
| **XGB-ROS** | 0.045 | 0.030 |
| **XGB-SMOTE** | 0.033 | 0.032 |

Table S3. The best-performing model for each evaluation metric. The 0.5 probability threshold was used for evaluating models based on threshold metrics.

| **Evaluation Metrics** | | **Best Model** |
| --- | --- | --- |
| **Threshold**  **metrics*** | SN | XGB-SMOTE/XGB-ROS |
|  | SP | XGB/LR |
|  | AC | XGB/LR |
| **Rank**  **metrics** | PR-AUC | XGB |
|  | ROC-AUC | XGB-SMOTE |
| **Probability**  **metric** | BS | XGB |
